# Papillary Renal Cell Carcinoma in a Functioning Kidney Allograft: A Graft-Preserving Strategy with Cryoablation and mTORi Conversion

**DOI:** 10.3390/life16091513

**Published:** 2026-09-11

**Authors:** Nicola Schiavone, Dario Troise, Marco Finati, Anna Ricapito, Oscar Selvaggio, Silvia Mercuri, Martina Pia Miracapillo, Luciana Antonia Cirolla, Ugo Giovanni Falagario, Francesca Sanguedolce, Barbara Infante, Giuseppe Carrieri, Giovanni Stallone, Gaetano Valerio Palella

**Affiliations:** 1 Department of Urology and Renal Transplantation, University of Foggia, 71122 Foggia, Italy; nic.sch96@gmail.com (N.S.); marco.finati@gmail.com (M.F.); annaricapito96@gmail.com (A.R.); oscarsel@libero.it (O.S.); giuseppe.carrieri@unifg.it (G.C.); gaetano.palella@gmail.com (G.V.P.); 2 Nephrology, Dialysis and Transplantation Unit, Advanced Research Center on Kidney Aging (A.R.K.A.), Department of Medical and Surgical Sciences, University of Foggia, 71122 Foggia, Italy; dario.troise@unifg.it (D.T.); smercuri@ospedaliriunitifoggia.it (S.M.); luciana.cirolla@unifg.it (L.A.C.); barbara.infante@unifg.it (B.I.); giovanni.stallone@unifg.it (G.S.); 3 Division of Renal Medicine and Baxter Novum, Department of Clinical Science, Intervention and Technology, Karolinska Instituted, 141 52 Stockholm, Sweden; 4 Department of Pathology, University of Foggia, 71122 Foggia, Italy; martinamiracapillo@gmail.com (M.P.M.); francesca.sanguedolce@unifg.it (F.S.); 5 Urology Unit, Department of Molecular Medicine and Surgery, Solna, Karolinska Institutet, 141 52 Stockholm, Sweden

**Keywords:** kidney transplantation, papillary renal cell carcinoma, cryoablation, mTOR inhibitors, graft preservation

## Abstract

**Background:** Renal cell carcinoma (RCC) arising in a kidney allograft is a rare but challenging condition, requiring a careful balance between oncologic control and preservation of graft function. Minimally invasive nephron-sparing approaches may represent a valuable treatment option in selected recipients. **Case Presentation:** We report the case of a 59-year-old kidney transplant recipient diagnosed with papillary RCC in the renal allograft during routine imaging follow-up. The patient underwent CT-guided percutaneous cryoablation, combined with conversion of immunosuppressive therapy to an mTOR inhibitor-based regimen. The procedure was completed successfully without complications, and graft function was preserved. At 3-year follow-up, no evidence of local recurrence or distant metastasis was detected, with stable renal function maintained throughout the observation period. **Conclusions:** This case highlights the feasibility and effectiveness of percutaneous cryoablation as a nephron-sparing treatment for RCC arising in a renal allograft. In carefully selected high-risk transplant recipients, this approach may provide durable oncologic control while preserving graft function.

## 1. Introduction

Kidney transplant recipients are at increased risk of malignancy as a consequence of altered immune surveillance mechanisms related to long-term immunosuppressive therapy. Renal cell carcinoma (RCC) occurs in approximately 0.7% of kidney transplant recipients and is significantly more frequent than in the general population. Most tumors arise in native kidneys, whereas RCC involving the renal allograft is rare (≈0.2%). Notably, papillary RCC represents one of the most common histologic subtypes reported in this rare setting [1]. Compared with clear cell RCC, localized papillary RCC is generally associated with a more favorable oncologic prognosis, lower metastatic potential, and excellent cancer-specific survival, making nephron-sparing strategies particularly attractive in carefully selected patients [2].

Management of RCC in kidney allografts remains challenging because oncologic control must be balanced against preservation of allograft function. Although transplantectomy and radical nephrectomy were historically considered standard treatment options, advances in nephron-sparing surgery and minimally invasive therapies, together with evolving strategies for tailoring immunosuppressive therapy, have progressively shifted management toward conservative approaches. Moreover, surgery in transplanted kidneys may be technically demanding because of altered anatomy, adhesions, and peri-graft fibrosis [3].

Percutaneous ablative techniques, including radiofrequency ablation, microwave ablation, and cryoablation, have emerged as attractive alternatives for selected patients with small renal masses arising in kidney allografts. Among these approaches, cryoablation offers several advantages, including real-time visualization of the ice ball, precise monitoring of the ablation zone, reduced damage to surrounding structures, and preservation of renal parenchyma. A recent systematic review demonstrated high technical success rates, low complication rates, and satisfactory graft functional preservation following ablative treatment of renal allograft neoplasms [4]. In parallel, optimization of immunosuppressive therapy has gained increasing attention in the management of post-transplant malignancies. Particularly, conversion to mammalian target of rapamycin inhibitors (mTORi) may represent a valuable adjunctive strategy due to their immunosuppressive and potential antineoplastic properties, although evidence supporting their use specifically in renal allograft RCC remains limited and largely derives from observational studies and case reports [5]. Nevertheless, evidence remains limited because of the rarity of these tumors and the scarcity of long-term follow-up data.

Herein, we report the successful treatment of papillary RCC arising in a kidney allograft using CT-guided percutaneous cryoablation and conversion to mTORi, with preservation of graft function and no evidence of local recurrence after 3 years of imaging follow-up.

## 2. Case Presentation

A 59-year-old man with end-stage renal disease secondary to IgA mesangial glomerulonephritis underwent deceased-donor kidney transplantation in March 2001 following Basiliximab induction and corticosteroids immunosuppressive therapy. His medical history was significant for hypertension, dyslipidemia, and hypothyroidism. Following transplantation, the patient was started on a maintenance immunosuppressive regimen consisting of tacrolimus, mycophenolate mofetil and corticosteroids. Renal function remained stable over the long term, with no significant changes in serum creatinine levels and a nadir serum creatinine (sCr) of 1.9 mg/dL.

In June 2023, during routine ultrasonographic follow-up, a nodular lesion was incidentally detected in the transplanted kidney. Therefore, abdominal magnetic resonance imaging (MRI) was performed, demonstrating a suspicious complex cystic lesion located at the lower pole of the renal allograft, measuring approximately 28 mm × 24 mm and initially classified as Bosniak II.

**Figure 1:**
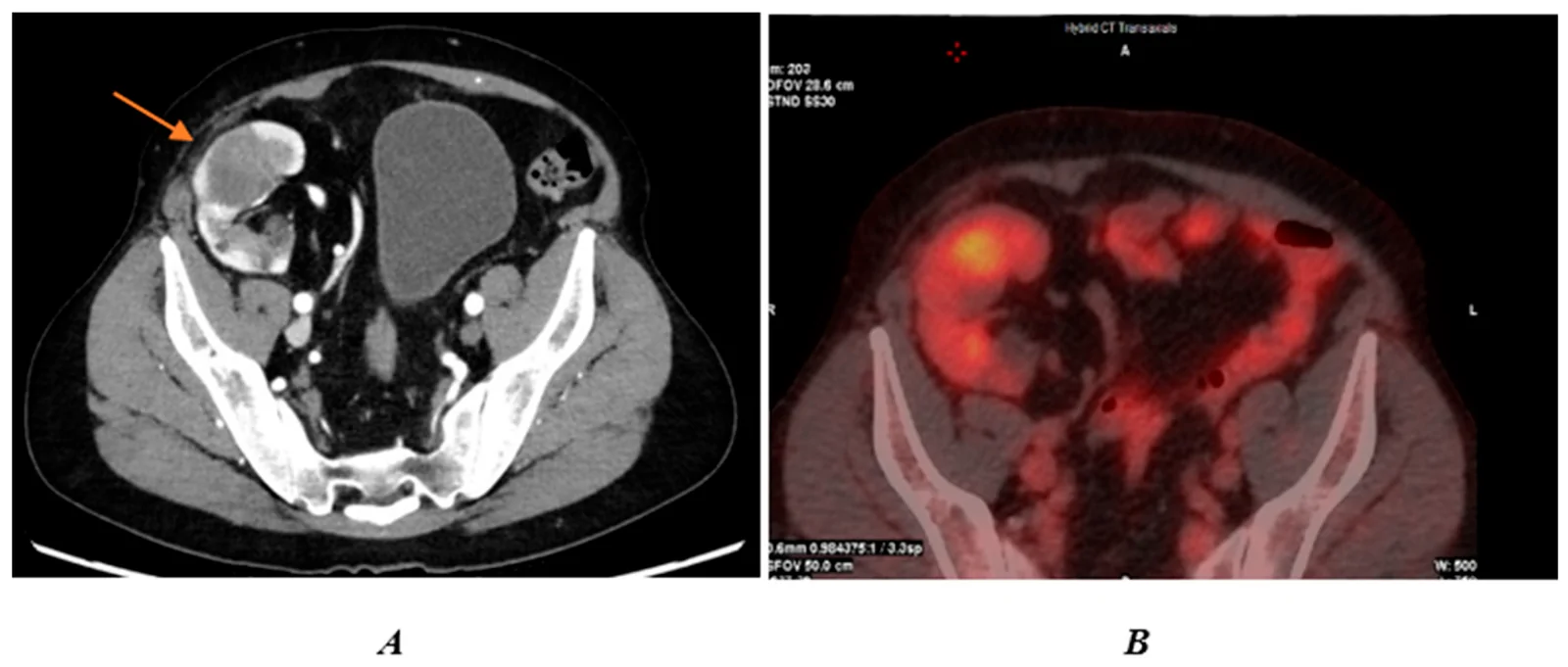
Multimodality imaging of papillary renal cell carcinoma in the kidney allograft prior to treatment. (**A**) Contrast-enhanced CT depicting the allograft lesion before CT-guided cryoablation. (**B**) ^18F-FDG PET/CT showing focal increased metabolic activity within the same lesion.

**Figure 2:**
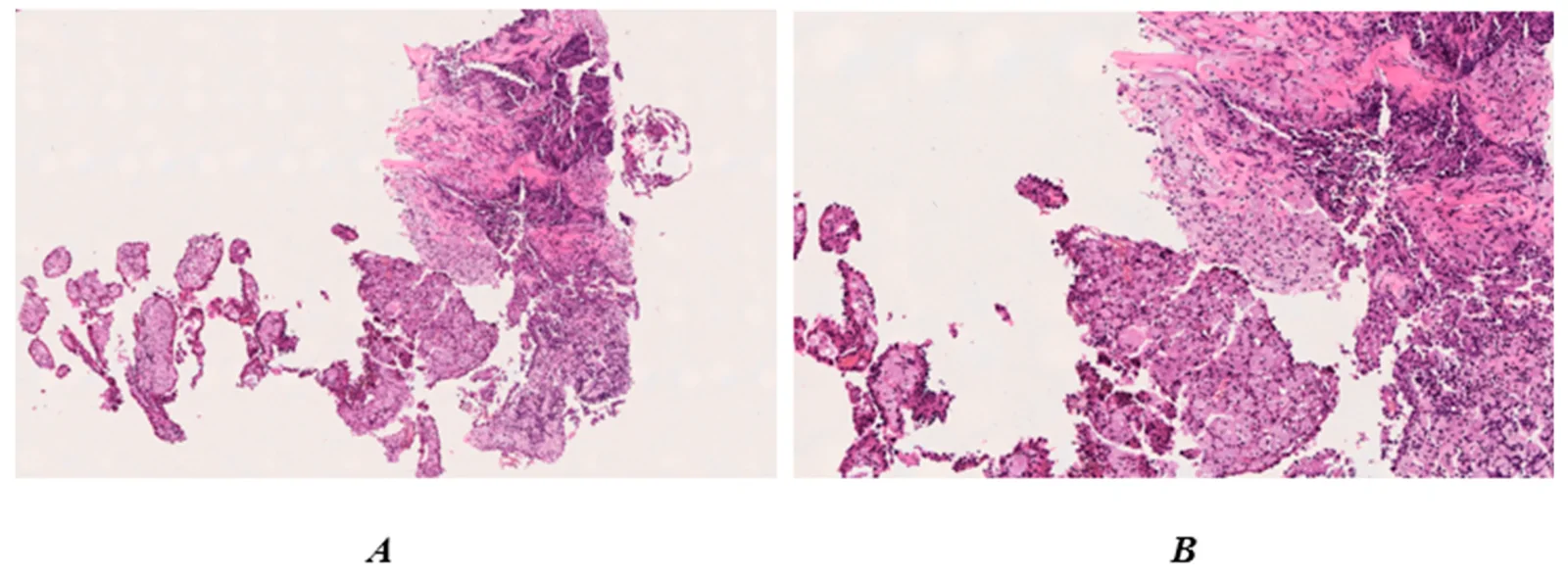
(**A**,**B**) Histopathological appearance of the renal mass biopsy specimen. (**A**) Low-magnification overview demonstrating a highly cellular neoplasm with fragmented, branching papillary and tubulopapillary architecture (Hematoxylin and Eosin stain, 40×). (**B**) Higher magnification reveals delicate fibrovascular cores lined by a single uniform layer of low-columnar cells with bland nuclei, accompanied by a rich infiltration of characteristic foamy histiocytes/macrophages within the papillary stalks (Hematoxylin and Eosin stain, 200×).

An initial strategy of close radiological surveillance was adopted. Follow-up contrast-enhanced abdominal computed tomography (CT) performed in November 2023 showed interval growth of the lesion, reaching 35 mm × 31 mm, with a predominant increase in the solid component. Subsequently, Fludeoxyglucose Positron Emission Tomography (18F-FDG PET/CT) demonstrated increased tracer uptake corresponding to the lesion [Figure 1]. Ultrasound-guided biopsy of the renal allograft mass was therefore performed, with histopathological examination consistent with papillary renal cell carcinoma with an immunophenotype of CAIX−, CK7+, AMACR+, and PAX8+, with a low proliferative index (Ki-67 ≤ 5%) [Figure 2]. At discharge following biopsy, serum creatinine was 1.9 mg/dL and blood urea nitrogen was 83 mg/dL. Following the diagnosis, the immunosuppressive regimen was optimized through conversion to an mTOR inhibitor-based strategy, with withdrawal of mycophenolate mofetil and minimization of tacrolimus therapy.

**Figure 3:**
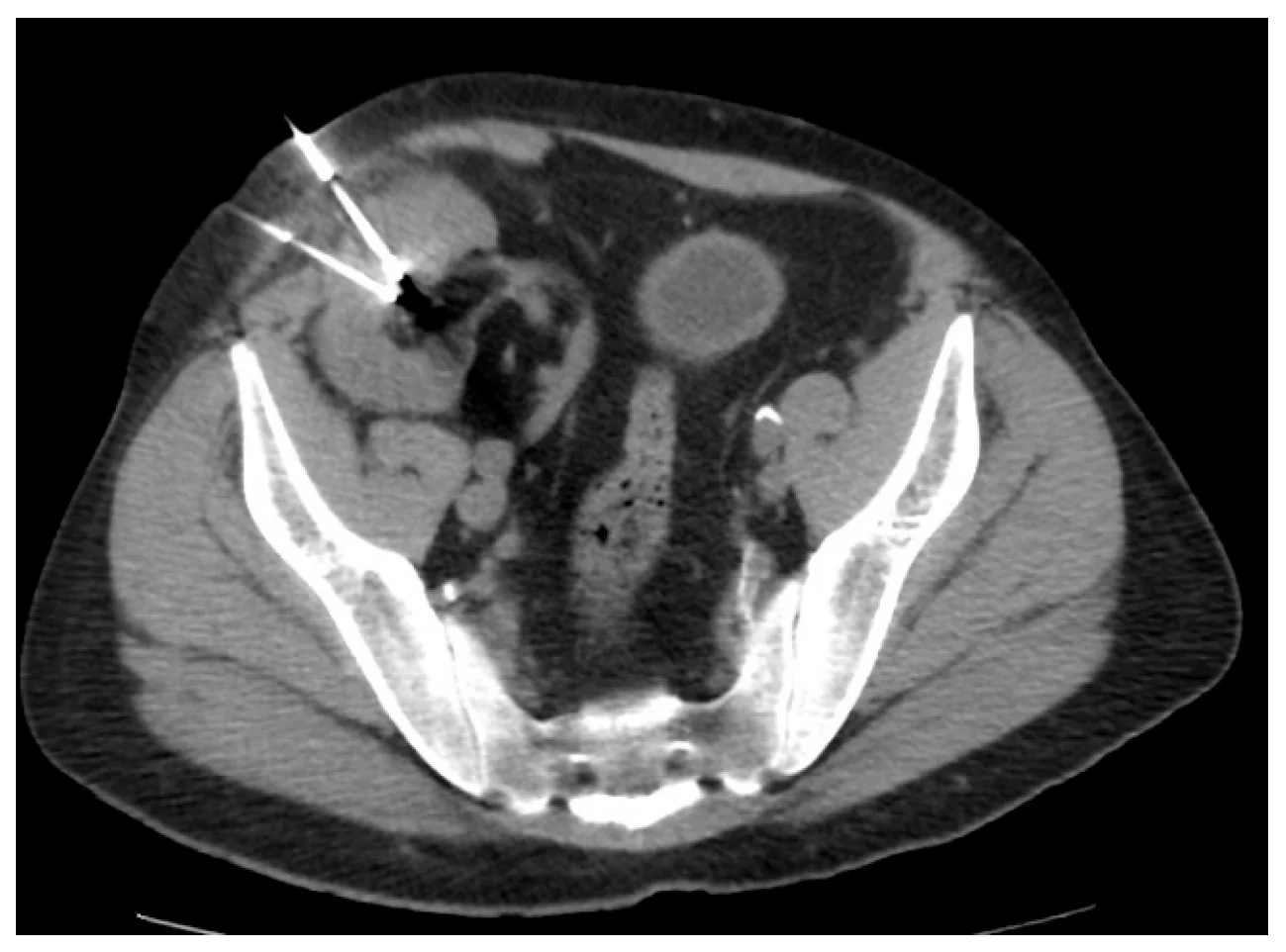
CT-guided percutaneous cryoablation of the renal allograft lesion. Intraprocedural CT image demonstrating cryoprobe placement within the tumor during cryoablation.

In December 2023, the patient underwent CT-guided percutaneous cryoablation under local anesthesia. The patient was informed about the diagnosis, the rationale for the proposed treatment, the potential risks and benefits of the available therapeutic options, and the possibility of disease progression. The patient understood and accepted the proposed treatment strategy, and written informed consent was obtained in accordance with applicable legislation. Three cryoprobes were positioned within the lesion (two 1.7-mm probes and one 2.4-mm probe), and two freeze–thaw cycles were performed. At the end of the procedure, the lesion appeared completely hypodense, consistent with complete treatment coverage [Figure 3]. No intraoperative or immediate post-procedural complications occurred. The postoperative course was uneventful, and renal graft function remained stable, with sCr of 1.7 mg/dL and blood urea nitrogen of 54 mg/dL at discharge.

**Figure 4:**
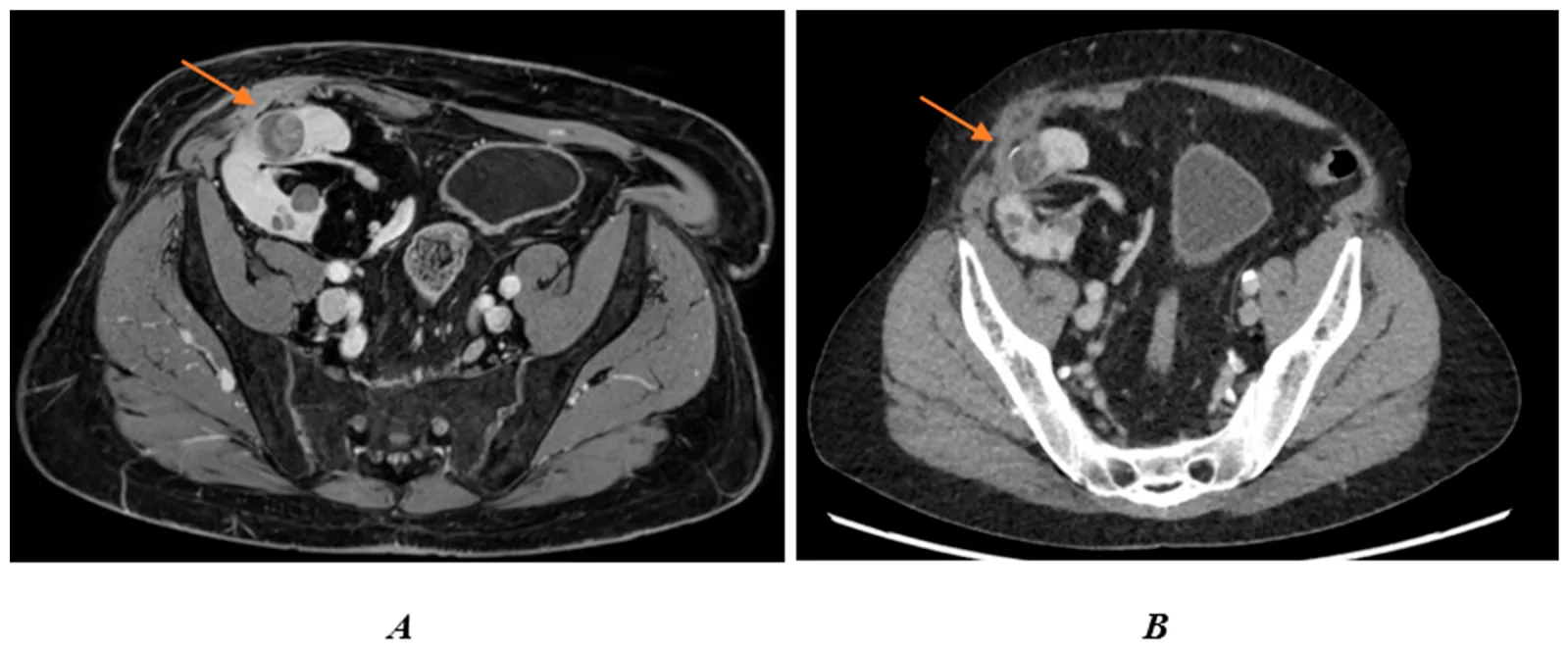
(**A**,**B**) Long-term radiological follow-up after CT-guided cryoablation. (**A**) MRI obtained 1 year after treatment showing post-ablation changes in the renal allograft lesion without residual disease. (**B**) Contrast-enhanced CT scan at 3-year follow-up confirming the absence of local recurrence.

**Figure 5:**
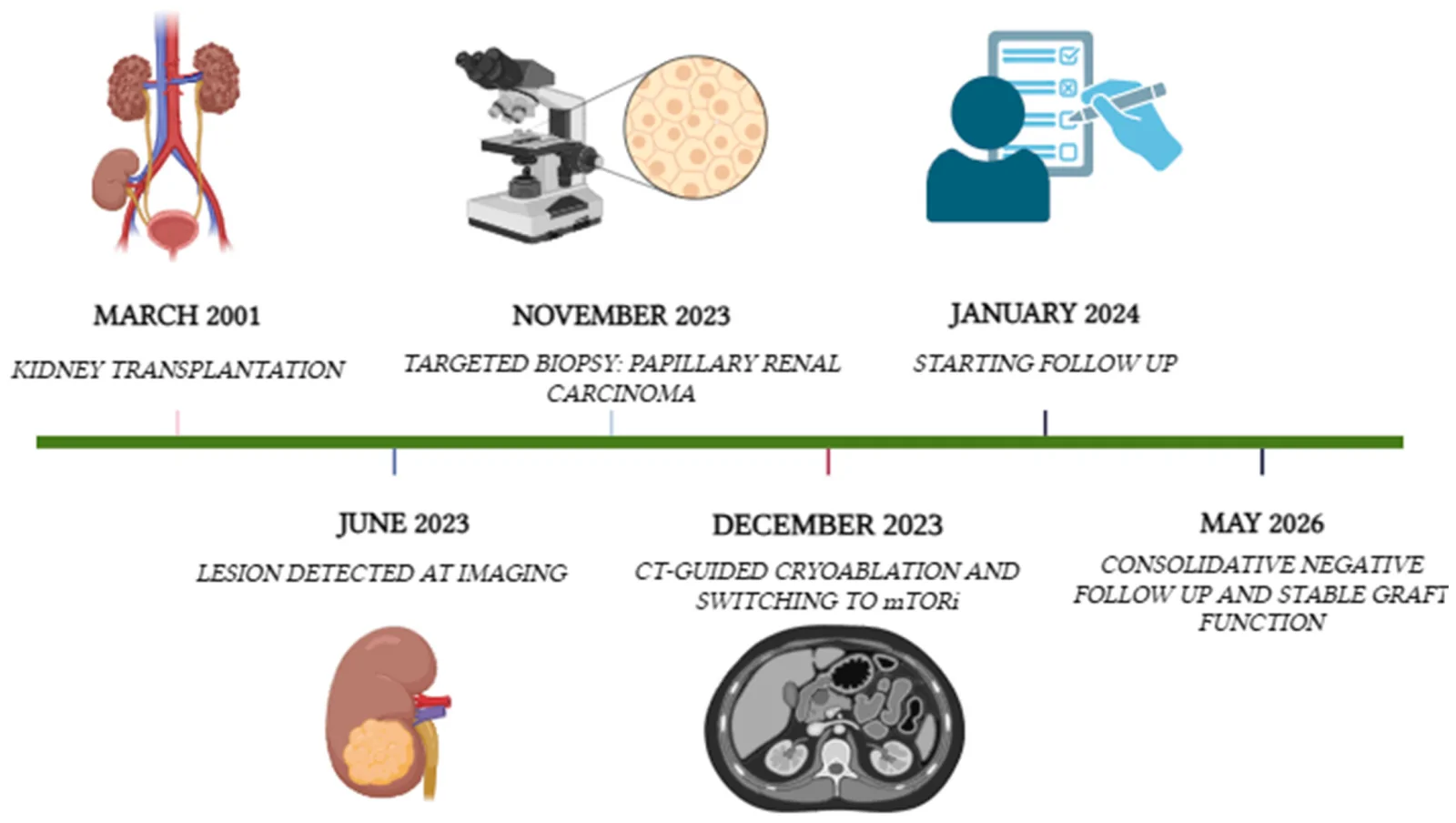
Timeline of the diagnostic work-up, therapeutic interventions, and long-term follow-up of a kidney transplant recipient with papillary renal cell carcinoma of the renal allograft treated with CT-guided cryoablation and mTOR inhibitor conversion (created with BioRender.com).

The patient was subsequently enrolled in a semestral imaging follow-up program with CT and MRI. No evidence of local recurrence or distant disease has been observed to date. In particular, the most recent CT scan performed at the end of 2025 demonstrated further dimensional reduction in the treated lesion (21 × 23 mm) without residual post-contrast enhancement, consistent with complete response after cryoablation [Figure 4]. Renal graft function remained stable during follow-up, with the latest sCr level measuring 2.0 mg/dL. In Figure 5 we reported the timeline of the diagnostic work-up, therapeutic interventions, and long-term follow-up.

## 3. Discussion

Management of renal masses arising in functioning kidney allografts remains particularly challenging because treatment decisions must balance oncologic control with preservation of graft function. This case illustrates the feasibility of a graft-preserving strategy combining CT-guided percutaneous cryoablation with optimization of immunosuppressive therapy for biopsy-proven papillary RCC. At the same time, it highlights the complexity of selecting patients who may benefit from intervention, an issue for which robust evidence and standardized recommendations are still lacking. Although Bosniak II cystic lesions are generally considered benign and do not require routine follow-up according to current guidelines, a strategy of close radiological surveillance was adopted because of the lesion’s occurrence in a renal allograft and the patient’s chronically immunosuppressed status [2,3,4,5,6]. In the present case, although the lesion was initially classified as Bosniak II and the low Ki-67 proliferative index (≤5%) suggested a lesion with potentially limited biological aggressiveness, interval imaging demonstrated an overall increase in lesion size and FDG-PET/CT showed increased tracer uptake. Moreover, although clear cell papillary renal cell tumor (ccpRCT) represents an important differential diagnosis for cystic renal tumours characterized by a generally indolent clinical behavior, the immunophenotype observed in our case (CAIX−, CK7+, AMACR+, PAX8+) was considered more consistent with papillary RCC particularly because the combination of CAIX negativity and AMACR positivity is not typical of ccpRCT [7]. The possibility of sampling-related misclassification cannot be completely excluded in a cystic lesion; however, the pathological findings were considered together with the clinical and radiological features in determining the therapeutic approach in order to achieve local tumour control while minimizing the risk of graft loss and return to dialysis.

Over the last decade, increasing evidence has supported the use of nephron-sparing strategies and minimally invasive ablative techniques for selected patients with localized allograft RCC. In particular, focal ablative therapies have emerged as attractive alternatives to transplantectomy, especially in patients with small renal masses and functioning grafts, allowing preservation of renal function while reducing perioperative morbidity. Several therapeutic strategies have been described, including nephron-sparing surgery and image-guided ablative techniques such as radiofrequency ablation (RFA), microwave ablation (MWA), and cryoablation [3,4,8]. While surgery may provide definitive oncological treatment, it can be technically challenging in the transplant setting, and graft nephrectomy inevitably results in loss of graft function. Minimally invasive ablative approaches therefore represent valuable graft-preserving alternatives in selected patients [4,8]. However, available evidence on ablative therapies in kidney allografts remains limited to case reports and small retrospective series. In a systematic review including 100 ablative procedures in 92 kidney transplant recipients, Favi et al. reported low perioperative morbidity, stable graft function in the majority of patients, and excellent local tumor control, supporting ablative therapies as a valid nephron-sparing alternative in selected cases [4]. In our case, a conservative approach was deliberately pursued in order to avoid graft loss and the consequent return to chronic dialysis. Similar to the experience reported by Barama et al., preservation of graft function represented a primary therapeutic goal in our patient, supporting the role of conservative strategies in selected cases of allograft RCC [9]. Notably, none of the studies included in the review provided detailed information regarding modifications of maintenance immunosuppressive therapy following tumor diagnosis or treatment, leaving the potential role of immunosuppression adjustment largely unexplored.

Cryoablation was preferred over other nephron-sparing strategies because of its superior visualization of the ablation zone through direct monitoring of the ice ball, allowing greater procedural control and potentially reducing the risk of damage to the collecting system and surrounding structures [8]. These characteristics may be particularly advantageous in renal allografts, where preservation of functional parenchyma is essential and anatomical constraints may increase the technical complexity of treatment.

CT guidance represented a key technical advantage in our case, allowing accurate probe placement and real-time visualization of the ice-ball margins throughout the procedure. In transplanted kidneys, which are typically located in the iliac fossa and may present altered anatomy because of previous surgery and peri-graft fibrosis, CT guidance may improve procedural precision and facilitate protection of adjacent structures. The ability to directly monitor the ablation zone is particularly relevant in nephron-sparing procedures aimed at preserving graft function [10].

Finally, the favourable outcome observed in our patient was likely related not only to cryoablation itself, but also to the integrated therapeutic strategy adopted. Following histological diagnosis, immunosuppressive therapy was converted to an mTOR inhibitor-based regimen. Beyond their immunosuppressive effect, mTOR inhibitor-based regimen has been proposed as a potential strategy to reduce tumor progression while maintaining graft function in transplant recipients with de novo malignancies [11]. Tacrolimus is a potent and widely used immunosuppressive drug that exerts its effects primarily through inhibition of calcineurin, thereby suppressing T-cell activation and cytokine transcription, including interleukin-2, and also modulate immune responses through the inhibition of costimulatory pathways [12]. In contrast, mTOR inhibitors exert antiproliferative and antiangiogenic effects through inhibition of the PI3K/Akt/mTOR pathway, a key regulator of tumour growth and progression. Clinical evidence of their antitumour activity has been reported in transplant recipients, particularly in non-melanoma skin cancers and Kaposi sarcoma, where conversion from calcineurin inhibitors to mTOR inhibitors has been associated with reduced tumour burden and recurrence [13,14]. In contrast, evidence for renal cell carcinoma in kidney transplant recipients remains limited and is mainly derived from small series and case reports [15]. Therefore, the favourable outcome observed in our patient may reflect the combined effect of local tumour control achieved by cryoablation and the potential antitumour activity of mTOR inhibition. Therefore, the combination of conservative image-guided cryoablation and immunosuppressive optimization may represent an effective multimodal strategy for selected patients with renal allograft malignancies, maximizing oncologic control while preserving graft survival.

Our case adds further evidence supporting the long-term efficacy of cryoablation in renal allograft tumors. The absence of recurrence after three years of follow-up, together with stable graft function, reinforces the encouraging preliminary data available in the literature and confirms the feasibility of CT-guided cryoablation in this high-risk population.

Nevertheless, several limitations should be acknowledged. First, as this is a single-case report, the findings cannot be generalized to the broader population of kidney transplant recipients with renal allograft tumors. Second, the favourable outcome observed in our patient was likely multifactorial and cannot be attributed solely to cryoablation, as the subsequent conversion to an mTOR inhibitor-based immunosuppressive regimen may also have contributed to disease control. Finally, although the three-year recurrence-free follow-up is encouraging, it does not exclude the possibility of late tumour recurrence, highlighting the need for continued long-term surveillance.

## 4. Conclusions

Papillary renal cell carcinoma arising in a functioning kidney allograft is a rare clinical entity for which standardized management strategies are lacking. This case supports that CT-guided cryoablation with conversion to an mTOR inhibitor-based immunosuppressive regimen may achieve durable oncologic control while maintaining allograft function in carefully selected patients. Moreover, this case highlights the importance of individualized decision-making through a multidisciplinary approach. Given the limited evidence currently available, additional studies and longer follow-up are needed to better define the role of ablative therapies and immunosuppressive optimization in the management of renal allograft malignancies.

## Data Availability

The data presented in this study are available on request from the corresponding author. The data are not publicly available.

## Abbreviations

The following abbreviations are used in this manuscript:

RCC	Renal Cell Carcinoma
CT	Computed Tomography
mTORi	Mammalian Target of Rapamycin Inhibitors
sCr	Serum Creatinine
MRI	Magnetic Resonance Imaging
18F FDG PET	Fluorodeoxyglucose Positron Emission Tomography
